# Baclofen in Refractory Dyspepsia With Chronic Belching: A Case Report

**DOI:** 10.7759/cureus.106942

**Published:** 2026-04-13

**Authors:** Mariana Rocha, Lisete Silva, Francisco Coelho

**Affiliations:** 1 Family Medicine, USF Egas Moniz de Ribadouro, ULS do Tâmega e Sousa, Penafiel, PRT; 2 Family Medicine, Departamento de Cuidados de Proximidade, ULS do Tâmega e Sousa, Penafiel, PRT

**Keywords:** baclofen, chronic belching, functional dyspepsia, mental health disorders, primary care

## Abstract

Dyspeptic symptoms associated with chronic belching may substantially affect quality of life and can be challenging to manage in clinical practice. We report the case of a 40-year-old nulligravid woman with depressive disorder, bilateral cochlear implants due to deafness, a hiatal hernia, and a previous pulmonary embolism. Her medications included desvenlafaxine, multiple benzodiazepines, mirtazapine, trazodone, and apixaban. She presented with an 18-month history of persistent belching, heartburn, early satiety, and intermittent dry cough, severely impairing her quality of life and leading to social isolation. Additionally, her symptoms intensified during anxiety and depressive episodes.

Physical examination was unremarkable, and upper gastrointestinal endoscopy revealed only a small hiatal hernia and mild gastropathy, with no evidence of *Helicobacter pylori* infection. Despite repeated medical evaluations and multiple therapeutic approaches, including proton pump inhibitors, sucralfate, domperidone, simethicone, and bilastine, symptoms persisted without significant improvement. Functional dyspepsia was therefore suspected, and treatment with baclofen 10 mg twice daily was initiated, resulting in significant clinical improvement and enhanced quality of life.

This report highlights the potential benefit of baclofen in patients with persistent belching and dyspeptic symptoms refractory to conventional therapy, particularly in the context of psychiatric comorbidity.

## Introduction

Functional dyspepsia affects approximately 16% of the population [1] and presents with postprandial fullness, early satiety, and epigastric pain or heartburn, often accompanied by chronic belching, without evidence of structural disease [2,3]. These symptoms significantly impact quality of life, resulting in increased healthcare utilization and, in some cases, social isolation. The etiology is multifactorial and includes gastric motility dysfunction, visceral hypersensitivity, and dysregulation of the brain-gut axis [4]. Identified risk factors include female sex, smoking, overweight status, anxiety or depressive disorders, and a history of gastroenteritis [5]. Proton pump inhibitors are typically used as first-line therapy; however, many patients remain refractory. Baclofen, a GABA_B_ receptor agonist, is considered a last-line treatment that has shown success in some cases [6]. We report a case of dyspepsia with chronic belching, refractory to conventional therapy, which was successfully treated with baclofen.

## Case presentation

The patient was a 40-year-old nulligravid woman who had a medical history of poorly controlled depressive disorder, bilateral cochlear implants for deafness, and a history of pulmonary embolism. Her regular medication regimen consisted of desvenlafaxine 150 mg and flurazepam 15 mg in the morning; clorazepate dipotassium 10 mg three times daily; clonazepam 0.5 mg; mirtazapine 15 mg and trazodone 100 mg at night; and apixaban 5 mg twice daily. She had no known drug allergies.

Over the preceding 18 months, the patient had repeatedly consulted her family physician for persistent belching that was unrelated to mealtimes and was associated with heartburn, intermittent early satiety, and occasional dry cough. These symptoms negatively affected her quality of life, eventually leading to social withdrawal. She reported that symptom worsening often coincided with periods of increased anxiety and depressive symptoms and denied anorexia, asthenia, unintentional weight loss, or gastrointestinal bleeding. On physical examination, the patient appeared in good general condition, with normal mucosal coloration and hydration. Her BMI was 28.9 kg/m², and the oral mucosa and abdominal examination were unremarkable. No alarm features were identified throughout the clinical course. Initial investigations included an upper gastrointestinal endoscopy performed in February 2024, which revealed a small hiatal hernia without surgical indication and mild congestive gastropathy, with negative testing for *Helicobacter pylori*. A therapeutic trial with omeprazole 20 mg once daily for one month was initiated, but there was no clinical improvement.

During the following year, the patient sought medical care multiple times in primary care, urgent care, and private consultations because of persistent symptoms. Several therapeutic approaches were attempted, including sucralfate, simethicone, bilastine, and domperidone, without resolution of symptoms. She was also advised on lifestyle modifications, including regular physical activity and dietary measures such as reducing the intake of carbohydrates, coffee, chocolate, and other potentially symptom-triggering foods; however, adherence to these recommendations was inconsistent, largely due to poorly controlled anxiety and depressive symptoms. In July 2025, in the absence of structural disease and given the persistence of symptoms, a diagnosis of functional dyspepsia was considered, and treatment with baclofen 10 mg twice daily was initiated. The diagnosis was clinical, and no relevant imaging or photographic documentation was available. At follow-up, the patient reported a marked reduction in the frequency and intensity of belching and dyspeptic symptoms, which became intermittent rather than occurring daily, resulting in a significant improvement in quality of life. Mild symptom fluctuations persisted over time, which the patient continued to associate with exacerbations of depressive symptoms.

## Discussion

Dyspepsia is a syndrome characterized by epigastric pain, postprandial fullness, and early satiety, and it may also be associated with chronic belching [2,3,7]. In about 80% of cases, no structural cause is identified for these symptoms [1]. Functional dyspepsia is a differential diagnosis to consider and is estimated to affect up to 16% of the population [8]. In pathophysiological terms, several authors consider it to be an entity closely related to mental health, as part of what they describe as the brain-gut axis. Thus, it is believed to result from alterations in motility, visceral hypersensitivity, and dysbiosis [4,5]. According to the Rome IV criteria, this condition is defined by the presence of at least one of the following symptoms, in the absence of an explanatory structural cause: postprandial fullness; early satiety; epigastric pain or burning, with or without chronic belching. Symptoms must have occurred at least six months before diagnosis and have persisted for the past three months [2,3,8].

**Figure 1:**
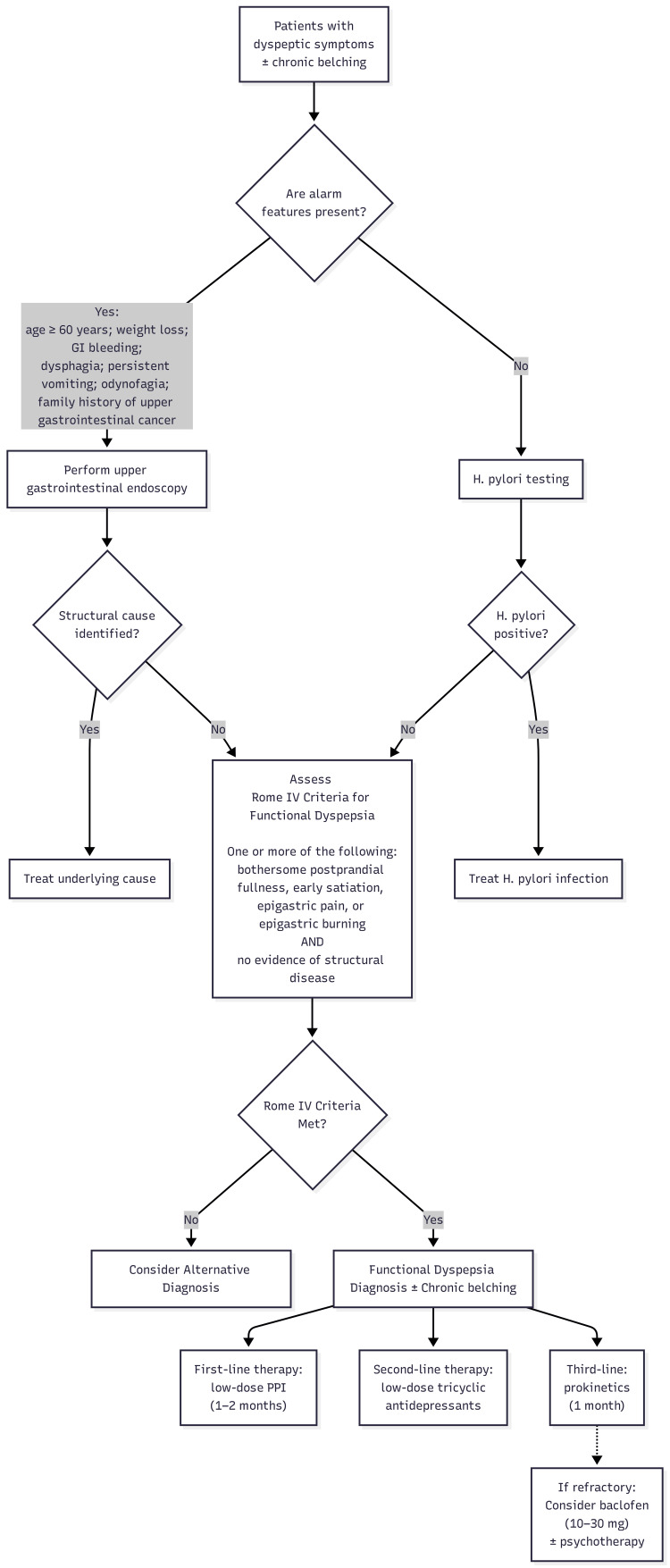
Diagnostic and therapeutic approach to functional dyspepsia with chronic belching The figure illustrates the stepwise diagnostic evaluation and therapeutic approach to patients with persistent dyspeptic symptoms and chronic belching, including exclusion of structural causes, assessment of alarm features, and consideration of treatment options for refractory cases GI: gastrointestinal; *H. pylori*: *Helicobacter pylori*; PPI: proton pump inhibitor Image created by the authors

Regarding risk factors, the literature highlights female sex, young age, smoking, chronic use of non-steroidal anti-inflammatory drugs, past *Helicobacter pylori* infection, even if eradicated, overweight and obesity, acute gastroenteritis, and psychiatric disorders such as major depressive disorder and anxiety disorders [4,5]. Functional dyspepsia is a diagnosis of exclusion; therefore, the approach to these patients involves excluding structural causes, including performing endoscopic studies if necessary, and considering warning signs for serious disease or neoplasia. All patients with suspected functional dyspepsia should undergo testing for *Helicobacter pylori* and be treated accordingly, as outlined in Figure 1 [6,9,10].

In practical terms, current scientific knowledge classifies management into three main therapeutic lines. If *Helicobacter pylori* infection is absent, first-line treatment with a low-dose proton pump inhibitor once daily on an empty stomach for one to two months is recommended. The following regimens are considered adequate: esomeprazole 20 to 40 mg, lansoprazole 15 to 30 mg, omeprazole 20 mg, pantoprazole 20 to 40 mg, or rabeprazole 10 to 20 mg. If the response is satisfactory, treatment should be maintained for six months and then gradually reduced to the minimum effective dose that maintains symptom control [11]. However, approximately one-third of patients do not respond to this approach. Therefore, as a second-line treatment, low-dose tricyclic antidepressants such as amitriptyline or nortriptyline 10 mg are suggested, with dose escalation as tolerated [9,10,12]. If symptoms persist, prokinetics are a third-line treatment, for example, metoclopramide at a maximum of 10 mg three times daily for up to one month [9,10,13].

Finally, baclofen, a GABA_B_ receptor agonist, may be a therapeutic option in selected cases. Baclofen increases the tone of the lower esophageal sphincter and suppresses its transient relaxations, thereby preventing reflux [14]. Although its use in functional dyspepsia is not recommended as a first-line treatment, several studies suggest that it may be beneficial for selected patients, especially when chronic belching and comorbid psychiatric disorders are present [15,16]. It is especially effective in patients with ruminative behaviours, aerophagia, or chronic belching. A dose of up to 30 mg daily may be used, although the lowest effective dose is recommended [14-16].

In addition, lifestyle measures should be considered a cornerstone of management and used as adjuncts to both pharmacological and psychological interventions. Regular physical activity should be encouraged. Dietary modifications may also be considered, including the reduction of symptom-triggering foods; a low-FODMAP diet can be suggested in selected cases, although robust evidence supporting dietary interventions in functional dyspepsia remains limited [17]. Given that it is a multifactorial condition with a strong association with psychiatric disorders, cognitive behavioural therapy is a therapeutic option to consider, including gut-brain behavioural psychotherapy. This is particularly successful when the symptoms are associated with periods of stress or worsening psychiatric illness [6,18]. The evolution of functional dyspepsia tends to be chronic and fluctuating, but it has not been shown to increase mortality [4].

In our patient, symptomatic worsening was associated with periods of increased anxiety and depressive symptoms, suggesting an interaction between psychosomatic factors and esophageal motility. Given this correlation between dyspeptic symptoms and mental disturbance, baclofen proved to be a successful therapy after the failure of first-line treatments described in the literature. This highlights the importance of an integrated and holistic approach in general and family medicine, with appropriate coordination between gastroenterology, psychiatry, and psychology for adequate management of these cases, especially when a significant change in the pattern of health service use is observed.

## Conclusions

This report highlights the importance of considering functional dyspepsia in patients with persistent upper gastrointestinal symptoms after structural causes have been excluded. Chronic belching may represent an underrecognized contributor to refractory dyspeptic symptoms and should be actively evaluated during clinical assessment. Repeated healthcare visits may also reflect inadequately controlled chronic symptoms and should prompt clinicians to reassess the diagnosis and consider alternative management strategies. In addition, the report underscores the relevance of a comprehensive biopsychosocial approach, particularly in patients with psychiatric comorbidities, as these may significantly influence symptom perception and response to treatment. In selected patients with prominent belching and persistent dyspeptic symptoms, baclofen may represent a useful therapeutic option for improving symptom control and overall quality of life.
